# Role of emotional control on anxiety and stress among cancer patients

**DOI:** 10.1002/cam4.70162

**Published:** 2024-09-20

**Authors:** Robert Jan Łuczyk, Kamil Sikora, Agnieszka Bodio, Marta Łuczyk, Monika Baryła‐Matejczuk, Agnieszka Wawryniuk, Katarzyna Sawicka, Agnieszka Zwolak

**Affiliations:** ^1^ Department of Internal Medicine and Internal Nursing, Faculty of Health Sciences Medical University of Lublin Lublin Poland; ^2^ Department of Long‐Term Nursing, Faculty of Health Sciences Medical University of Lublin Lublin Poland; ^3^ Institute of Psychology and Human Sciences WSEI University Lublin Poland

**Keywords:** anxiety, cancer, coping with stress, emotional control, health, oncology, psycho‐oncology

## Abstract

**Objective:**

The article aims to assess the role of stress and anxiety in relation to the level of emotional control among cancer patients. Currently cancer ranks second, after cardiovascular disease, as the most common cause of death. Moreover, it is predicted that in the coming years, cancer will become the leading cause of death worldwide. This is due to the extended lifespan of the population and also to the presence of carcinogenic factors in the surrounding environment. The emergence of cancer is a significant stressor that affects individuals in diverse ways, leading to cognitive, behavioral, and emotional consequences. In line with the adopted aim, emotional issues are the chosen area of exploration in this article.

**Methods:**

The study included 102 patients. The differences between the patients' results according to various scales and the results produced by the validation group data were examined using one‐sample *t*‐tests. The relationships between the quantitative variables were determined using Spearman's rho coefficients, and the relationships between the quantitative and qualitative variables were verified using Kruskal‐Wallis tests.

**Results:**

The participants exhibited higher anxiety suppression levels than individuals in the normalization group. They sought emotional support more frequently than the average person in the population, turned to religion, engaged in other such activities, lived in denial more often, discontinued activities, and displayed a sense of humor less frequently. The more frequently they controlled their anger, the less they sought emotional and instrumental support, catharsis, and attempted to accept the situation and cease being active. Additionally, controlling anxiety, sadness, and depression coexisted with self‐blame, denial, and compensatory actions.

**Conclusions:**

Cancer patients face intense anxiety. Emotional and instrumental support, along with the ability to express and manage emotions, are crucial for these patients, especially within the context of facing the challenge of cancer. Finding constructive ways to express strong and difficult emotions prevents their accumulation and reduces the need for emotional suppression. Preventive actions should be oriented toward supporting the emotional competencies of patients.

## Graphical abstract

Cancer patients face intense anxiety. Emotional and instrumental support, along with the ability to express and manage emotions, is crucial for these patients, especially in the context of a challenging illness like cancer. Finding constructive ways to express strong and difficult emotions such as anger and fear prevents their accumulation and reduces the need for control.

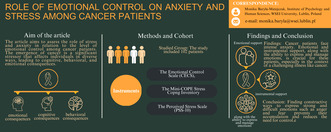

## 1 INTRODUCTION

Cancer diseases are a significant and multidimensional issue worldwide.^1^, ^2^, ^3^ Moreover, there is an upward trend in the incidence of such diseases. In Poland, cancer is the second leading cause of death after cardiovascular disease^4^ and may become the leading one.^5^ The increasing occurrence of this phenomenon makes it worthwhile to examine cancer not only from a medical perspective but also from a psychological one. It is normal for patients to experience intense emotional reactions in response to the challenging new situations which serious illness present (cf.^6^), hence, due to the prevalence of a multitude of unpleasant feelings associated with cancer, terms like “cancer distress,” and “cancer‐related stress”^7^ have been used for years to describe the nature of the adverse experiences in patients. Certain aspects of emotion regulation are indicated as the key to coping with emotional burdens, and particularly with emotion control as individuals attempt to manage the generation, experience, or expression of emotions and/or their emotional reactions.^8^, ^9^ Research has confirmed the importance of this psychological feature in cancer patients^10^ because the ways in which individuals are able to achieve emotional control have implications for their health and general well‐being.^11^, ^12^

Previous studies suggest that a high level of resilience and a low degree of emotional control appear to be protective attributes, while a high degree of emotional control has been identified as a risk factor for mental health.^13^ Moreover, current research demonstrates the existence of significant relationships between anxiety, depression, and patient age (such issues occur more frequently in older individuals) among cancer patients. Significant associations have also been found between anxiety, depression, the type of cancer, and the type of treatment.^14^ Nearly half of women with early‐stage breast cancer experienced depression, anxiety, or both within a year after diagnosis, and the lack of intimate and confiding support was also a predictive factor for longer episodes of depression and anxiety.^15^, ^16^

The direction and intensity of emotional processes are influenced by individual predispositions. Emotional states are highly complex phenomena, they involve physiological processes, brain events (triggering cognitive‐evaluative functions), feelings (subjective experiential components), and behavioral phenomena (cf.^17^). Emotions can also play a significant motivating role in our actions.^18^ According to the interactive concept of stress,^19^ it is the relationship between an individual and their environment, which the individual interprets as burdensome, surpassing their resources, and threatening their well‐being. Therefore stressful situations have specific characteristics related to human functioning in the environment and concern various responses to stimuli.^19^, ^20^ Disease is a potent source of stress that can influence an individual's behavior, and experiencing stress can be a risk factor in falling ill.^21^

Cancer is considered to be one of the most stressful categories of disease,^22^ and the emergence of cancer is a potent stressor that affects individuals in diverse ways, leading to cognitive, behavioral, and emotional consequences (cf.^23^). Stress, as well as future‐oriented anxiety, are related to the functioning of the immune, hormonal, and nervous systems. The disease may become a cause of unfavorable changes across all aspects of human life, significantly disrupting the daily lives of the patient and their loved ones, and resulting in significant distress.^24^ Additionally, it is known that cancer patients who experience anxiety and depression differ in the perceived intensity of their symptoms (cf.^25^) Variables potentially linked to the level of anxiety reveal a complex picture of mutual relationships. Various socio‐demographic variables are associated with anxiety and depression: gender—women undergoing cancer treatment reported higher levels of anxiety and depression; age—older individuals at the start of their cancer treatment are more prone to depression than anxiety; social support—providing support to prostate cancer patients in the first month after diagnosis leads to a reduction in the intensity of their emotional difficulties.^26^, ^27^ These findings indicate the need for screening the intensity of stress and related anxiety in patients in order to adequately support them and enhance their quality of life.^28^

Taking into account the research conducted to date, the aim of this research was to assess the intensity of the stress, anxiety and the resulting stress‐coping strategies, as well as assessing the role of emotional control concerning the perception of anxiety and stress among cancer patients.

## 2 MATERIALS AND METHODS

The study included patients diagnosed with cancer, who were receiving treatment at the Clinic of Pneumonology, Oncology, and Allergology, as well as the Oncology and Chemotherapy Department at the Independent Public Clinical Hospital No. 4 in Lublin. The research was conducted from March 2021 to May 2021. A total of 102 patients who were provided with research tools participated in the study. The patients participating in the study gave their written consent for its execution and were informed about its purpose and their anonymity.

### 2.1 Instruments

The following research tools were used in this research:

1. A self‐constructed questionnaire—consisting of 16 questions concerning demographic and medical variables;
2. The Emotional Control Scale (CECS) by Maggie Watson and Seven Greer (Polish adaptation: Zygfryd Juczyński).^29^ CECS comprises 21 statements (three subscales consisting of seven questions each) that allow one to obtain a general score related to emotional control, as well as three subscales related to anger control, depression control, and anxiety control. The reliability of the scale for the Polish adaptation was assessed by estimating its internal consistency and absolute stability. The Cronbach's alpha coefficient for internal consistency was 0.87 for the overall emotional control index (0.80 for anger control, 0.77 for depression control, 0.78 for anxiety control). The stability coefficients of the subscales ranged from 0.36 to 0.49 and were lower than those of the original version.
3. The Mini‐COPE Stress Coping Inventory by C. S. Carver (Polish adaptation: Z. Juczyński, N. Ogińska‐Bulik).^30^ The inventory is used to study adults (both healthy and ill individuals) and comprises 28 statements constituting 14 strategies (two statements for each strategy). The method is often used to measure dispositional coping, that is, assessing typical ways of reacting to and experiencing particularly stressful situations. The internal consistency of the Polish version of Mini‐COPE was established based on a study of 200 individuals aged 25–60 years. The split‐half reliability was 0.86. Diagnostic validity was assessed by correlating the Mini‐COPE results with the Mini‐MAC scale, it was designed to study coping strategies in cancer patients, as well as predicting the intensity of posttraumatic stress symptoms in a group of mothers of children being treated for leukemia.
4. The Perceived Stress Scale (PSS‐10) by S. Cohen, T. Kamarck, R. Mermelstein (Polish adaptation: Z. Juczyński and N. Ogińska‐Bulik),^30^ consisting of 10 questions relating to various subjective experiences associated with personal problems and life events. The internal consistency of Cronbach's alpha was 0.86. The split‐half reliability based on a study conducted twice within a 2‐day interval was 0.90, while within a 4‐week interval it was found to be 0.72.5 The State–Trait Anxiety Inventory (STAI) by C. D. Spielberger, R. L. Gorsuch, R. E. Lushene^31^ (Polish adaptation: C.D. Spielberger, J. Strelau, M. Tysarczyk, K. Wrześniewski).^32^ The questionnaire has two subscales comprising 20 items each. The first subscale assesses anxiety as a state, while the second assesses anxiety as a trait. The internal consistency of both subscales is high. The validity of both scales has been confirmed in many studies.

### 2.2 Statistics

Differences between the results obtained by the participants using various scales and also the results of the validation group data were examined using one‐sample *t*‐tests. Assumptions regarding the normality of distribution and the size of the potential differences between the obtained distribution and the normal distribution were verified beforehand. The relationships between the quantitative variables were examined using Spearman's rho coefficients. The nonparametric correlation coefficient was chosen due to the presence of outliers in two‐dimensional distributions of variables (examined using scatter plots). The relationships between the quantitative and qualitative variables were verified using the Kruskal‐Wallis tests. This nonparametric method had the advantage of the lack of a normal distribution in the compared variables with significant fluctuations in the sizes of the compared groups (sometimes very small *n* < 10). The effect size for the observed differences was expressed using the eta‐squared or Cohen's d measures.

### 2.3 Studied group

A total of 102 individuals participated in the study. Table 1 presents the demographic characteristics of the group. The largest group of participants belonged to the age range of 61–70 years. The majority of participants had a secondary education, with approximately 20% having primary, vocational, or higher education. The place of residence divided the participants into nearly equal parts. The majority of individuals were married (over 60%) and identified themselves as believers (98.0%).

**TABLE 1:** Demographic characteristics of the studied group.

Variable		Frequency	Percentage
Male/Female	Woman	59	57.84%
	Man	43	42.16%
Age	<31	3	2.94%
	31–40	5	4.90%
	41–50	17	16.67%
	51–60	20	19.61%
	61–70	42	41.18%
	>70	15	14.71%
Education	Primary	21	20.59%
	Vocational	20	19.61%
	Secondary	40	39.22%
	Higher	21	20.59%
Place of residence	Rural area	52	50.98%
	Urban area	50	49.02%
Marital status	Single	8	7.84%
	Married	63	61.76%
	Divorced	10	9.80%
	Widowed	21	20.59%
Occupational activity	Studies	1	0.98%
	Works	24	23.53%
	Unemployed	18	17.65%
	Retired	59	57.84%
Attitude toward faith/religion	Believer	100	98.04%
	Nonbeliever	2	1.96%

In the majority of cases, the patients had a malignant tumor (88.2%), while every tenth participant was burdened with a benign tumor (Table 2). Most frequently, the respondents suffered from lung or digestive system cancers. The majority of respondents had been ill for no more than a year. They accounted for over 60% of the entire sample. The most common treatment methods among the participants were chemotherapy and surgical treatment. Treatment‐related decisions were made jointly by the respondents and the doctor for the most part (56.9%). In assessing their level of knowledge about the disease, the majority of the respondents considered themselves well‐informed (88.2%) and family support was felt by 92.2% of respondents, while its absence was reported by 7.8%.

**TABLE 2:** Characteristics of the illness.

Variable		Frequency	Percentage
Tumor type	Benign	12	11.76%
	Malignant	90	88.24%
Location of the tumor	Lungs	32	31.37%
	Breasts	10	9.80%
	Skin	9	8.82%
	Digestive system	27	26.47%
	Urinary‐reproductive system	7	6.86%
	Reproductive organ	8	7.84%
	Head and neck	4	3.92%
	Soft tissue	1	0.98%
	Bones	3	2.94%
	Central nervous system	1	0.98%
Duration of the illness	Up to 6 months	33	32.35%
	7–12 months	35	34.31%
	13–24 months	24	23.53%
	over 24 months	10	9.80%
Treatment methods	Chemotherapy	53	51.96%
	Radiation therapy	9	8.82%
	Surgical treatment	41	40.20%
	Immunotherapy	0	0.00%
	Hormone therapy	0	0.00%
	Combined treatment	27	26.47%
Subjective evaluation of treatment	Very good	10	9.80%
	Good	39	38.24%
	Fair	45	44.12%
	Poor	8	7.84%
Impact on treatment‐related decisions	Decisions largely depend on me	8	7.84%
	Decisions are made jointly with the doctor	58	56.86%
	Most decisions are made by the doctor	28	27.45%
	All decisions are made by the doctor	8	7.84%
Self‐assessment of knowledge about the illness	Well‐informed	90	88.24%
	Uninformed	12	11.76%
Sources of information about the illness	From the doctor	93	91.18%
	From friends	15	14.71%
	From the internet	26	25.49%
	From scientific materials	8	7.84%
	From TV, radio, newspapers	10	9.80%
Family support	Yes	94	92.16%
	No	8	7.84%

## 3 RESULTS

### 3.1 Level of emotional control among patients with cancer

In the first stage of the analysis, an examination of the obtained results regarding emotional control in the group of cancer patients was conducted. The average score obtained by the participants was 52.06 points with a standard deviation of 11.08. The lowest level of emotional control was 30, while the highest was 83 points. The Shapiro–Wilk test did not indicate any statistically significant deviation of the distribution of results in the overall emotional control scale from a normal distribution (SW = 0.981; *p* = 0.151). Low skewness and kurtosis coefficients also suggest a lack of significant deviations (SKEW = 0.228; KURT = −0.082). In order to determine the level of emotional control in the studied group, the results obtained in the individual scales were compared with those of the normalization group for CECS. One‐sample *t*‐tests were applied for this purpose. The results are presented in Table 3. Since the distribution of participants based on gender did not differ between the validation and the study groups *χ*2 = 0.344; *p* = 0.557, the entire participant group was simultaneously compared to the validation group without gender division through the calculation of weighted means for the validation group's results.

**TABLE 3:** Level of emotional control in the group of individuals with cancer as compared to the normalization group of CECS.

Emotional control scale	*N*	M	*SD*	Tested value	*t*	*df*	*p*	D
Anger control	102	16,51	4,33	16,09	0,980	101	0,329	0,097
Depression control	102	17,05	4,04	16,87	0,447	101	0,655	0,044
Anxiety control	102	18,50	4,11	17,67	2040	101	0,044	0,202
Overall emotional control	102	52,06	11,08	50,62	1311	101	0,193	0,130

Abbreviations: df, degrees of freedom; d, Cohen's d effect size; M, Mean; n, number of observations; p, test probability; SD, Standard deviation; t, Student's *t*‐test result.

The conducted analysis revealed only one statistically significant, weak relationship between anxiety control and being diagnosed with cancer. The participants exhibited higher anxiety control than individuals from the normalization group for CECS. No statistically significant differences between the groups were found in the other scales of emotional control.

### 3.2 Emotional control and stress coping strategies

In the next stage of the analysis, the relationships between the level of emotional control among the participants and the coping strategies they used to deal with stress were examined (Table 4).

**TABLE 4:** Coping strategies used to mitigate stress used by the participants.

Stress coping strategy	M	*SD*	Me	Min	Max	S‐W	*p*
Active coping	2.059	0.577	2.00	0.00	3.00	0.246	0.000
Planning	2.083	0.585	2.00	0.50	3.00	0.253	0.000
Seeking instrumental support	2.064	0.688	2.00	0.00	3.00	0.247	0.000
Seeking emotional support	2.230	0.566	2.00	1.00	3.00	0.227	0.000
Blaming	1.137	0.809	1.00	0.00	3.00	0.185	0.000
Turning to religion	1.941	0.794	2.00	0.00	3.00	0.304	0.000
Positive reframing	1.672	0.603	2.00	0.00	3.00	0.236	0.000
Venting	1.755	0.511	1.50	0.00	3.00	0.221	0.000
Acceptance	1.858	0.646	2.00	0.00	3.00	0.197	0.000
Denial	1.088	0.665	1.00	0.00	3.00	0.219	0.000
Distracting oneself	2.123	0.560	2.00	0.00	3.00	0.198	0.000
Cessation of activity	0.907	0.579	1.00	0.00	2.50	0.250	0.000
Alcohol use	0.627	0.624	1.00	0.00	2.00	0.274	0.000
Sense of humor	0.559	0.752	0.00	0.00	3.00	0.281	0.000

Abbreviations: M, Mean; n, number of observations; p, test probability; SD, Standard deviation; S‐W, Shapiro–Wilk test result; t, Student's *t*‐test result.

The Shapiro–Wilk tests conducted revealed statistically significant differences between the distributions obtained in the all mini‐COPE scales and the normal distribution. The participants coping with stress rarely resorted to humor, alcohol consumption, cessation of activity, denial, self‐blame, while frequently utilizing positive reframing, engagement in other activities, acceptance, discharge, turning to religion, seeking emotional and instrumental support, planning, and active coping strategies. In order to compare the frequency of using a given stress coping strategy between the average individuals in the population and the surveyed group, a series of one‐sample *t*‐tests were applied. The results are presented in Table 5.

**TABLE 5:** Stress coping strategies in the surveyed group as compared to the validation group using the mini‐COPE scale.

Stress Coping Strategy	*N*	M	*SD*	Tested value	T	*df*	*p*	D
Active coping	102	2.06	0.58	1.87	3.304	101	0.001	0.327
Planning	102	2.08	0.58	1.89	3.339	101	0.001	0.331
Seeking instrumental support	102	2.06	0.69	1.56	7.392	101	0.000	0.732
Seeking emotional support	102	2.23	0.57	1.66	10.181	101	0.000	1.008
Blaming	102	1.14	0.81	1.2	−0.783	101	0.435	0.078
Turning to religion	102	1.94	0.79	0.85	13.882	101	0.000	1.375
Positive reframing	102	1.67	0.60	1.67	0.026	101	0.979	0.003
Venting	102	1.75	0.51	1.01	14.723	101	0.000	1.458
Acceptance	102	1.86	0.65	1.78	1.216	101	0.227	0.120
Denial	102	1.09	0.67	0.63	6.956	101	0.000	0.689
Distracting oneself	102	2.12	0.56	1.34	14.103	101	0.000	1.396
Cessation of activity	102	0.91	0.58	0.58	5.701	101	0.000	0.564
Alcohol use	102	0.63	0.62	0.37	4.166	101	0.000	0.412
Sense of humor	102	0.56	0.75	0.82	−3.507	101	0.001	0.347

Abbreviations: df, degrees of freedom; d, Cohen's d effect size; M, Mean; n, number of observations; p, test probability; SD, Standard deviation; t, Student's *t*‐test result.

The conducted analyses revealed several statistically significant differences between the studied group and the validation group of mini‐COPE. The participants sought emotional support, turned to religion, discharged their emotions, engaged in distracting activities, and lived in denial more frequently as compared to the average person in the population. They also ceased to be active and denied it more often than individuals in the normalization group. Additionally, they consumed alcohol more frequently, while using humor less often than individuals in the normalization group. Table 6 presents the relationships between stress coping strategies and the level of emotional control. These were examined using the Spearman's rank correlation coefficient.

**TABLE 6:** Emotional control and stress coping strategies.

Stress coping strategy	Anger control		Depression control		Anxiety control		General emotional control	
	Rho	*p*	Rho	*p*	rho	*p*	Rho	*p*
Active coping	−0.082	0.415	−0.106	0.289	−0.134	0.180	−0.132	0.186
Planning	0.019	0.850	0.014	0.887	0.006	0.952	0.008	0.936
Seeking instrumental support	**−0.269**	**0.006**	**−0.335**	**0.001**	**−0.282**	**0.004**	**−0.335**	**0.001**
Seeking emotional support	**−0.326**	**0.001**	**−0.378**	**0.000**	**−0.330**	**0.001**	**−0.385**	**0.000**
Blaming	0.123	0.217	**0.242**	**0.014**	**0.311**	**0.001**	**0.232**	**0.019**
Turning to religion	0.024	0.810	−0.097	0.333	−0.036	0.719	−0.045	0.651
Positive reframing	0.098	0.329	−0.113	0.257	−0.166	0.096	−0.064	0.522
Venting	**−0.289**	**0.003**	**−0.246**	**0.013**	−0.073	0.468	**−0.211**	**0.034**
Acceptance	**0.269**	**0.006**	0.146	0.144	0.101	0.311	**0.204**	**0.039**
Denial	0.104	0.298	0.181	0.069	**0.222**	**0.025**	0.173	0.083
Distracting oneself	0.076	0.446	0.132	0.184	**0.269**	**0.006**	0.177	0.076
Cessation of activity	**0.202**	**0.042**	0.128	0.200	0.141	0.157	0.177	0.075
Alcohol use	−0.102	0.306	0.034	0.737	0.006	0.954	−0.031	0.757
Sense of humor	0.146	0.143	0.105	0.292	0.128	0.201	0.128	0.198

*Note*: Bold values indicates *p* < 0.05.

Abbreviations: p, test probability; rho, Spearman's rho coefficient.

The conducted analyses revealed several statistically significant relationships, most of which were weak. Moderate correlations were observed between seeking emotional support and all dimensions of emotion suppression (negative correlation), as well as seeking instrumental support and the control of depression as well as general emotion control. Blaming oneself and the control of anxiety also showed a moderate negative correlation.

With regard to anger control, seeking emotional and instrumental support and discharge, they all showed negative correlations, while acceptance and a cessation of activity showed positive correlations. Similarly, negative correlations were observed between depression and sadness control and seeking instrumental and emotional support as well as discharge. A positive correlation was observed with blaming oneself. Similarly, the more the patient controlled their anxiety, the less likely they were to seek emotional and instrumental support, and the more likely they were to blame themselves, engage in denial, or other negative thought processes.

### 3.3 Emotion control and a sense of stress in the group of individuals with cancer

The aim of the next stage of the analysis was to verify the relationship between the level of emotional control among the participants and their perceived level of stress. The PSS‐10 questionnaire was used to measure this stress. The average score obtained by the participants in the stress perception scale was 22.20 points with a standard deviation of 5.41. The lowest level of stress intensity was 7, while the highest was 35. The Shapiro–Wilk test indicated statistically significant differences between the distribution of the scores in the PSS‐10 stress perception scale and the normal distribution (SW = 0.970; *p* = 0.021). However, these differences were not substantial, as indicated by the low skewness and kurtosis coefficients (SKEW = −0.449; KURT = −0.188). In order to relate the results obtained by the participants to the overall population, the scores obtained by the participants were compared using a one‐sample t‐test with the mean scores obtained by the validation group for PSS‐10. The results obtained are presented in Table 7.

**TABLE 7:** The intensity of stress in the group of participants as compared to the validation group in PSS‐10.

Scale	*n*	M	*SD*	Tested value	*t*	*df*	*p*	D
Perceived stress	102	22.20	5.41	16.62	10.406	101	0.000	1.030

Abbreviations: df, degrees of freedom; d, Cohen's d effect size; M, Mean; n, number of observations; p, test probability; SD, Standard deviation; t, Student's *t*‐test result.

The conducted analyses revealed a significantly higher level of experienced stress among the participants as compared to the average person in the population.

Table 8 presents the results of the correlation analysis of the perceived stress levels of the participants.

**TABLE 8:** The perceived stress levels and emotional control of the patient.

Emotional control scale	rho	*p*
Anger control	−0.136	0.172
Depression control	−0.044	0.659
Anxiety control	−0.004	0.968
General emotional control	−0.081	0.417

Abbreviations: p, test probability; rho, Spearman's rho coefficient.

The conducted analysis did not reveal any statistically significant correlations between the sense of stress experienced by the subjects and their level of emotional control.

### 3.4 Emotional control and the occurrence of anxiety in a group of individuals with cancer

The aim of the next stage of the analysis was to verify the relationship between the level of emotional control of the participants and their experience of anxiety, as well as their overall tendency to experience anxiety: anxiety as a currently experienced emotion (state) and anxiety propensity (trait).

The average score obtained by the participants on the scale of currently experienced anxiety was 51.88 points with a standard deviation of 10.33. The lowest intensity was 20, while the highest was 73. The Shapiro–Wilk test indicated statistically significant differences between the distribution of scores on the scale of current state anxiety and a normal distribution (SW = 0.972; *p* = 0.027). However, these differences were not substantial, as indicated by the low skewness and kurtosis coefficients (SKEW = −0.533; KURT = 0.105).

The average score obtained by the participants on the scale of anxiety propensity was 52.16 points with a standard deviation of 8.46. The lowest intensity was 26, while the highest was 68. The Shapiro–Wilk test indicated statistically significant differences between the distribution of scores on the scale of current state anxiety and a normal distribution (SW = 0.958; *p* = 0.002). However, these differences were not substantial, as indicated by the skewness and kurtosis coefficients (SKEW = −0.771; KURT = 0.508), although some left‐skewness of the results was evident (a relative prevalence of higher scores over lower ones).

The participants' results were converted to a stanine scale (ranging from 1 to 10, with a mean of 5.5 for the population and a standard deviation of 2). The results of the participants in stanines were then compared with the results of an average individual in a population of a similar age and gender to the participants, using the Student's t‐test. The results are presented in Table 9.

**TABLE 9:** Level of anxiety in the examined group as compared to the validation group of the State–Trait Anxiety Inventory (STAI).

Scale	*n*	M	SD	Tested value	*t*	*df*	*p*	D
Anxiety as a state	102	8.09	1.90	5.50	13.753	101	0.000	1.362
Anxiety as a trait	102	7.27	1.97	5.50	9.070	101	0.000	0.898

Abbreviations: df, degrees of freedom; d, Cohen's d effect size; M, Mean; n, number of observations; p, test probability; SD, Standard deviation; t, Student's *t*‐test result.

The conducted analyses showed a significantly higher experience of anxiety in the examined group as well as a significantly higher tendency to experience anxiety as compared to the average person in the population. Table 10 presents the results of the correlation analysis between the subjects' sense of anxiety and their level of emotional control.

**TABLE 10:** Subjects' sense of anxiety and emotional control.

Emotional control scale	Anxiety as a state		Anxiety as a trait	
	Rho	*p*	Rho	*p*
Anger contol	−0.182	0.067	−0.142	0.156
Depression contol	**−0.229**	**0.020**	−0.122	0.222
Anxiety control	−0.188	0.058	−0.101	0.311
General emotional control	**−0.226**	**0.023**	−0.137	0.171

Abbreviations: p, test probability; rho, Spearman's rho coefficient.

The conducted analysis revealed statistically significant, yet weak correlations between the currently experienced state of anxiety and the control of depression, as well as overall emotional control. The higher the intensity of the anxiety experienced by the participants, the more difficult it was for them to control their depression and also their emotions in general.

## 4 DISCUSSION

In the described study it was shown that the cancer patients exhibited higher levels of anxiety suppression as compared to the normalization group. The research did not find any differences between the cancer patients and the normalization group in the remaining emotional control scales, thereby indicating that the control of sadness and depression did not differ significantly. In other Polish studies conducted by Glińska, Krzemińska, Lewandowska, Miller, Dziki, and Dzik in 2014,^33^ the level of emotional control and the need for social support among women with breast cancer were assessed. The research revealed that the majority of the examined patients exhibited relatively high levels of emotional control overall. In comparison with the current study, the research by Galińska et al. showed similar results in terms of the emotional control level scale.

On the one hand, suppression may be an appropriate method for reducing anxiety‐related distortions in time perception.^34^ On the other hand, research has shown^35^ that suppression strategies for regulating anxiety arousal are the least effective of the three established strategies (as compared to reappraisal, acceptance). It is disadvantageous both in terms of the cost of cognitive attention^36^ the course of the disease itself and the treatment process.^37^, ^38^, ^39^

The studies also show that in coping with stress, patients sought emotional support, and used both constructive methods (they turned to religion, released their emotions by engaging in alternative activities) and destructive methods which related to the use of stimulants. The strategies used by the group of oncology patients have also been confirmed by other studies,^40^ which paid special attention to the fact that strategies aimed at improving emotionality may be particularly beneficial.^41^

By addressing socio‐cultural factors, in the subsequent Polish studies conducted,^42^ the emotional control of patients being treated for cancer was also tested. These studies did not find a relationship between the duration of the illness, demographic variables, and the degree of emotional control.^42^ Another study that analyzed emotional control among individuals undergoing oncological treatment (for breast cancer) was conducted by Zdończyk.^43^ Furthermore, some studies indicated that women exhibited the greatest tendency to suppress emotions in the area of anxiety,^43^ with a higher level of perceived stress while better controlling emotions such as anger, depression, and anxiety,^44^ which aligned with the results of the current study.

### 4.1 Conclusions

The research conducted led to the tentative conclusion that patients with cancer deal with higher levels of anxiety and also tend to suppress this emotion more intensely. These results concern the studied sample and may provide the basis for further verification in other groups of patients. The research group was found to utilize positive revaluation strategies, make plans, substitute their usual activities for alternatives, practice acceptance, engage in emotional release, turn to religion and seek emotional and instrumental support, they also used active coping techniques when dealing with challenges. They consumed alcohol more frequently, but used humor less often. Their anger control was found to be inversely proportional to their tendency to seek emotional and instrumental support and emotional release, while it was positively correlated with acceptance and ceasing previously normal activities.

The analyses conducted demonstrated a greater tendency in the studied cancer patients to experience anxiety as compared to the average person in the population. Furthermore, the higher the intensity of the anxiety experienced by the participants, the more difficult it was for them to control their depression and also to regulate their emotional state overall. The results obtained indicate the presence of a significant psychological burden on the studied cancer patients. These preliminary results require more research to confirm this tendency in cancer patients but it may also be assumed that this group of patients is diverse in terms of coping strategies. This may suggest the need for diverse communication and education in the field of effective and efficient forms of emotion regulation support and coping strategies. This strategy should include certain elements of competence which are supported by cognitive‐behavioral strategies and intervention programs for group risks that can be developed and transferred into guidelines designed to support the quality of life of patients.

### 4.2 Clinical implications

In conclusion, it's important to note that anxiety can be paralyzing, but it also presents an opportunity for a positive reaction – sufficient motivation to take action (including adhering to medical recommendations and cooperating with medical staff). This type of anxiety can be harnessed as a means of coping with cancer.^45^ Actively coping with cancer is associated with both a personal struggle and the search for emotional and instrumental support by individuals who are ill. Research confirms (including^46^) that actively coping with cancer has an impact on achieving a higher quality of life, extending survival time, it even has an impact on the manifestation of milder symptoms. The specific schedule for adopting coping styles with cancer depends on the time that has passed since diagnosis and treatment, and therefore it varies accordingly. Providing psychological care and conducting screening for the perceived intensities of stress, anxiety, and the resulting coping strategies in difficult situations can significantly enhance the mental and physical quality of life for oncology patients.

### 4.3 Study limitations and future research

In the realm of future research concerning the level of emotional control among patients with cancer, it is recommended to conduct studies with a larger number of participants. It would be useful to classify the studied individuals not only based on the duration of their cancer but also to take into account the degree of its advancement (cf.^46^). This is an important consideration, as the duration of the disease does not determine its stage, and cancers are often diagnosed at an advanced stage of their development. It's highly likely that a stronger relationship exists between this variable and emotional control.

Another area worthy of further exploration is the type of cancer that the patient is suffering from. It should be borne in mind that the study did not explore the relationship between anxiety, the progression of cancer and the prognosis for recovery. Further analyses should continue to explore this area and should also be undertaken to verify whether treatment or intervention in the case of cancer has an impact on the anxiety and depression states of patients, as well as exploring whether treatment or intervention in the case of anxiety has an impact on the progression of cancer. Controlling the relevant variables or designing a model in which variables have sufficient influence to moderate cancer progression could significantly increase the applied value of the research.

## AUTHOR CONTRIBUTIONS

**Robert Jan Łuczyk:** Conceptualization (lead); formal analysis (equal); investigation (equal); methodology (equal); project administration (equal); resources (equal); software (equal); supervision (equal); validation (equal); writing – original draft (equal). **Kamil Sikora:** Data curation (equal); formal analysis (equal); funding acquisition (equal); investigation (equal); methodology (equal); project administration (equal); resources (equal); software (equal); validation (equal); visualization (equal); writing – original draft (equal). **Agnieszka Bodio:** Investigation (equal); software (equal); validation (equal); visualization (equal); writing – original draft (equal). **Marta Łuczyk:** Investigation (equal); validation (equal); writing – original draft (equal). **Monika Baryła‐Matejczuk:** Supervision (equal); validation (equal). **Agnieszka Wawryniuk:** Data curation (equal); funding acquisition (equal); investigation (equal); writing – original draft (equal). **Katarzyna Sawicka:** Formal analysis (equal); funding acquisition (equal); investigation (equal); methodology (equal); project administration (equal); resources (equal); software (equal); validation (equal); visualization (equal). **Agnieszka Zwolak:** Data curation (equal); funding acquisition (equal); investigation (equal).

## FUNDING INFORMATION

This research received no external funding.

## CONFLICT OF INTEREST STATEMENT

The authors declare that there is no conflict of interest.

## ETHICS STATEMENT

The research was carried out as part of the activities of the Medical University of Lublin and the University Hospital in Lublin. Due to the regulations adopted by the university, research questionnaires are used as part of the work of the university hospital (educational and scientific value), and research on human subjects using medical instruments and biological data must have the support of the Bioethics Committee. The research project along with the full methodology used was approved by the Committee operating at the Faculty of Health Sciences of the Medical University of Lublin and exempted from the need to apply for the consent of the bioethics committee due to the selection of standardized tools and the survey nature of the research.

## CONSENT

The study was conducted in accordance with the Declaration of Helsinki. Informed consent was obtained from all subjects involved in the study. Written informed consent was obtained from the participants.

## Data Availability

The data that support the findings of this study are available on request from the corresponding author [M.B‐M]. The data are not publicly available due to certain restrictions (they contain information that could compromise the privacy of research participants).
